# Effectiveness of exercise as an intervention for internet addiction in adolescents: a meta-analysis

**DOI:** 10.7717/peerj.19999

**Published:** 2025-09-29

**Authors:** Luqiu Wang, Yonghuan Chen, Jin Ho Cho, Humin Yang, Jae Chul Seo

**Affiliations:** 1 School of Sport Science, Nantong University, Nantong, China; 2 Department of Marine Sports, Pukyong National University, Busan, Republic of South Korea; 3 Department of Fisheries Education, Pukyong National University, Busan, Republic of South Korea; 4 Department of Psychology, Fuyang Normal University, Anhui, China

**Keywords:** Internet addiction, Exercise intervention, Adolescents, Meta-analysis

## Abstract

**Background:**

Internet addiction, particularly among adolescents, has become a pressing global concern, impacting psychological well-being and daily functioning. This meta-analysis aims to evaluate the effectiveness of exercise interventions in mitigating symptoms of internet addiction among adolescents.

**Methods:**

A comprehensive literature search was conducted across PubMed, Web of Science (WoS), Cochrane Library, Embase, Scopus, and China National Knowledge Infrastructure (CNKI) databases up to January 1, 2025. The search yielded 266 records, of which nine studies involving 654 adolescents met the inclusion criteria. The quality of the included studies was assessed using the Cochrane Risk of Bias tool. Meta-analyses, subgroup analyses, and sensitivity analyses were conducted using Review Manager 5.4 and Stata 17.0. Publication bias was evaluated using Egger’s tests. This study is registered with PROSPERO (CRD42025632958).

**Results:**

The random effects model revealed that exercise interventions significantly reduced internet addiction scores compared to the control group (standardized mean difference (SMD) = −1.11, 95% CI [−1.60 to −0.62], *p* < 0.000). Subgroup analyses indicated that the type of exercise, duration, frequency, and intensity of interventions did not significantly contribute to heterogeneity. Sensitivity analysis confirmed the stability of these findings. Tests for publication bias yielded non-significant results (Egger’s test, *p* = 0.226). Exercise-based interventions demonstrate significant efficacy in reducing symptoms of internet addiction among adolescents.

**Conclusions:**

These findings underscore the importance of integrating physical activity into therapeutic strategies for managing internet addiction. The findings of this study will be useful for healthcare workers and adolescents with Internet addiction. However, due to the high heterogeneity of the included literature, these findings should be interpreted with caution.

## Introduction

Over the past decade, the internet and its associated digital platforms have become deeply integrated into the daily lives of adolescents. While this phenomenon has created new opportunities for access to information and social interaction, it has also introduced risks of behavioral dysregulation, such as internet addiction (IA) or specific forms of mobile phone dependency (Lu et al., 2025). IA refers to a chronic or recurring psychological dependence resulting from prolonged and excessive internet use. It is characterized by intense cravings for internet engagement and an inability to control online behaviors, often accompanied by increased tolerance, withdrawal symptoms, and compulsive usage (Méndez et al., 2024). Adolescence is marked by rapid psychological development and instability, making this age group particularly susceptible to excessive internet use and, consequently, a high-risk period for IA (Marin, Nuñez & De Almeida, 2021).

International research indicates that the overall prevalence of IA among adolescents is approximately 14%, with a rising trend in recent years—for example, increasing from 6% in 2012 to between 14% and 24% by 2022 (Yan et al., 2024). IA is associated with a range of psychological and physiological problems, including anxiety, depression, sleep disturbances, and deficits in cognitive control. Specifically, the physiological consequences of IA include reduced muscle strength, decreased endurance, dysregulation of physiological functions, weakened immune response, and obesity (Reed et al., 2015; Yıldız et al., 2024). On the psychological level, studies have shown that IA can impair individuals across both cognitive and non-cognitive domains. Cognitive impairments include declines in intellectual functioning, reduced attention span, and slowed thinking (Paterna et al., 2024). Non-cognitive impairments are manifested through increased levels of anxiety, depression, social withdrawal, and even suicidal ideation (Nikolic et al., 2023). These issues have been further exacerbated during the COVID-19 pandemic, with prevalence rates in some regions reportedly reaching as high as 36.7% (Li et al., 2021).

Given the significant threat that IA poses to individual health, the American Psychiatric Association has classified it as a potential emerging mental disorder and has emphasized the need for further research into its etiology, stability, and specific impacts on health and behavior (Przybylski, Weinstein & Murayama, 2017). At present, interventions for IA primarily include pharmacological treatments, behavioral therapies, group counseling, social support, and family-based interventions (Zhang et al., 2022). Although these approaches have demonstrated varying levels of effectiveness, theoretical and practical inconsistencies across disciplinary domains have hindered the integration of intervention strategies. As a result, current treatment methods remain fragmented, with limited variety and suboptimal effectiveness.

Physical exercise, as an evidence-based approach to improving both physical and mental health, has increasingly gained attention as a potential intervention for IA. Exercise not only directly addresses the physiological mechanisms underlying addiction by enhancing neural function, improving physical fitness, and regulating the secretion of dopamine and endorphins, but also indirectly mitigates psychological dependence by promoting emotional stability through increased self-esteem and self-confidence (Costa et al., 2019; Eather et al., 2023). Furthermore, the social and recreational dimensions of physical activity can serve as functional substitutes for certain aspects of internet use, such as reducing loneliness, fostering a sense of achievement, and facilitating social interaction (Ahn, EB & Kang, 2023). This functional complementarity positions physical exercise as a highly feasible and promising intervention strategy for individuals affected by IA. Other researchers have put forward similar views, arguing that students’ lifestyles can be improved by making them aware of the health risks associated with unhealthy lifestyles (Moscatelli et al., 2023). This has inspired efforts to reduce the severity of IA among adolescents by increasing their physical exercise and enhancing their metacognition.

Although existing research has demonstrated that physical activity has a significantly positive effect on alleviating IA among adolescents, the current body of literature exhibits considerable heterogeneity in intervention design, sample characteristics, assessment tools, and research methods. This variability reduces the comparability of findings and leads to inconsistent conclusions, with some studies also presenting a risk of bias. For example, a school-based physical activity intervention conducted among Italian adolescents indicated that regular participation in coordination training and team sports not only contributed to improvements in exercise motivation and sleep quality but also significantly reduced the risk of IA, with gender differences playing a notable moderating role (Greco et al., 2024). However, the study was observational in nature and lacked the capacity to establish causal inferences regarding the intervention’s effects.

On the other hand, systematic reviews and meta-analyses focusing on university students have shown that exercise interventions produce moderate to large effect sizes in reducing IA and alleviating associated psychological symptoms such as anxiety, loneliness, and depression. However, these studies also emphasize that the optimal combination of intervention type, frequency, and intensity remains to be clearly established (Yan et al., 2024). In addition, a network meta-analysis of 89 randomized controlled trials found that, among various intervention approaches, exercise as a standalone intervention yielded more favorable overall outcomes than traditional psychological methods such as cognitive behavioral therapy and family-based interventions, particularly for adolescent populations (Zhou et al., 2024). Nonetheless, the authors noted that due to the varying quality of the original studies, the conclusions require further validation through high-quality research.

In summary, although there is a preliminary consensus on the effectiveness of exercise interventions in the prevention and treatment of IA, considerable uncertainty and scholarly disagreement remain regarding the specific intervention mechanisms, applicable scope, and implementation standards. Therefore, this study aims to employ systematic review and meta-analysis methods to synthesize existing high-quality literature, determine the overall effect size of exercise interventions on adolescent internet addiction, and further examine the potential moderating effects of key variables such as intervention type, frequency, intensity, and duration. This research is expected to provide more scientific, standardized, and generalizable evidence-based support for clinical interventions, school-based health education, and public policy development targeting adolescent internet addiction.

## Methods and Materials

### Inclusion criteria

This meta-analysis were performed in accordance with the Preferred Reporting Items for Systematic Reviews and Meta-Analyses (PRISMA) guidelines, utilizing the Patient, Intervention, Control, Outcome, and Study design (PICOS) framework to establish the criteria for literature screening. This study has been registered in PROSPERO (No. CRD42025632958).

Patient:

The study was conducted on adolescents with IA who participated in an exercise intervention treatment.

Intervention:

The intervention implemented in the experimental group consisted of exercise, with no specific restrictions imposed on the duration, intensity, or frequency of the activity, allowing for a broad range of exercise protocols to be included.

Control:

The control group received usual care, including public education, medication, or no intervention.

Outcome:

The outcome is the level of IA for effect sizes combined for randomized controlled experiments.

Study design:

Only studies with a randomized controlled trials design were included.

### Exclusion criteria

1. Control participants received exercise as part of the intervention during the trials.
2. Studies for which data are not available and for which outcome are not relevant.
3. Duplicate records identified across multiple databases.
4. Studies lacking full-text access, such as conference abstracts. Systematic reviews and meta-analyses were also excluded.
5. Non-peer-reviewed papers, gray literature, or dissertations.

### Literature retrieval strategy

Extensive searches were conducted across multiple databases, including PubMed, Web of Science, Scopus, the Cochrane Library, Embase, and CNKI by Yonghuan Chen and Jin Ho Cho. The search encompassed all available records from the inception of each database through January 1, 2025. To identify relevant studies, a comprehensive strategy combining controlled vocabulary (*e.g.*, Medical Subject Heading (MeSH) terms) and free-text keywords was employed. The search terms included expressions such as “Internet Addiction Disorder,” “Internet Addiction,” “Smartphone Addiction,” and “Gaming Disorder,” as well as exercise-related terms including “Exercise Therapy,” “Rehabilitation Exercise,” “Resistance Training,” and “Endurance Training”.

### Literature screening and data extraction

The literature screening was conducted independently by Yonghuan Chen and Jin Ho Cho, who assessed study eligibility based on predefined inclusion and exclusion criteria. Upon completion of the data extraction, the two researchers cross-verified the collected information. In cases of disagreement, Humin Yang was consulted to mediate the discussion and make the final decision. Key data from the eligible studies were systematically extracted and recorded, including: (1) the first author’s name and year of publication; (2) sample characteristics of both the control and experimental groups; (3) specific intervention measures and their intensity in both groups; (4) intervention duration; and (5) outcome measures. To ensure objectivity, any discrepancies during the screening or extraction process were resolved through discussion with a third researcher until consensus was reached. When inconsistencies arose in the extracted data, they were rechecked against the original sources for accuracy. If no errors were identified, the data were directly included in the final analysis.

### Statistical analysis

Statistical analyses were conducted using Review Manager version 5.4 and Stata version 17.0. The Cochrane Risk of Bias tool was employed to assess the methodological quality of the included studies. The standardized mean difference (SMD) were calculated to account for variations in measurement scales and mean differences across studies. Heterogeneity was evaluated using the *I*^2^ statistic, with values greater than 50% indicating substantial heterogeneity. In cases of significant heterogeneity, a random-effects model was applied, and subgroup analyses were performed to explore potential sources of variation. When heterogeneity could not be explained by identifiable factors, a descriptive analysis was conducted as an alternative. Publication bias was assessed using Egger’s test, with a *p*-value above 0.05 indicating no significant bias. To ensure the robustness and reliability of the results, sensitivity analyses were conducted using the leave-one-out method.

## Results

### Retrieve results

**Figure 1:**
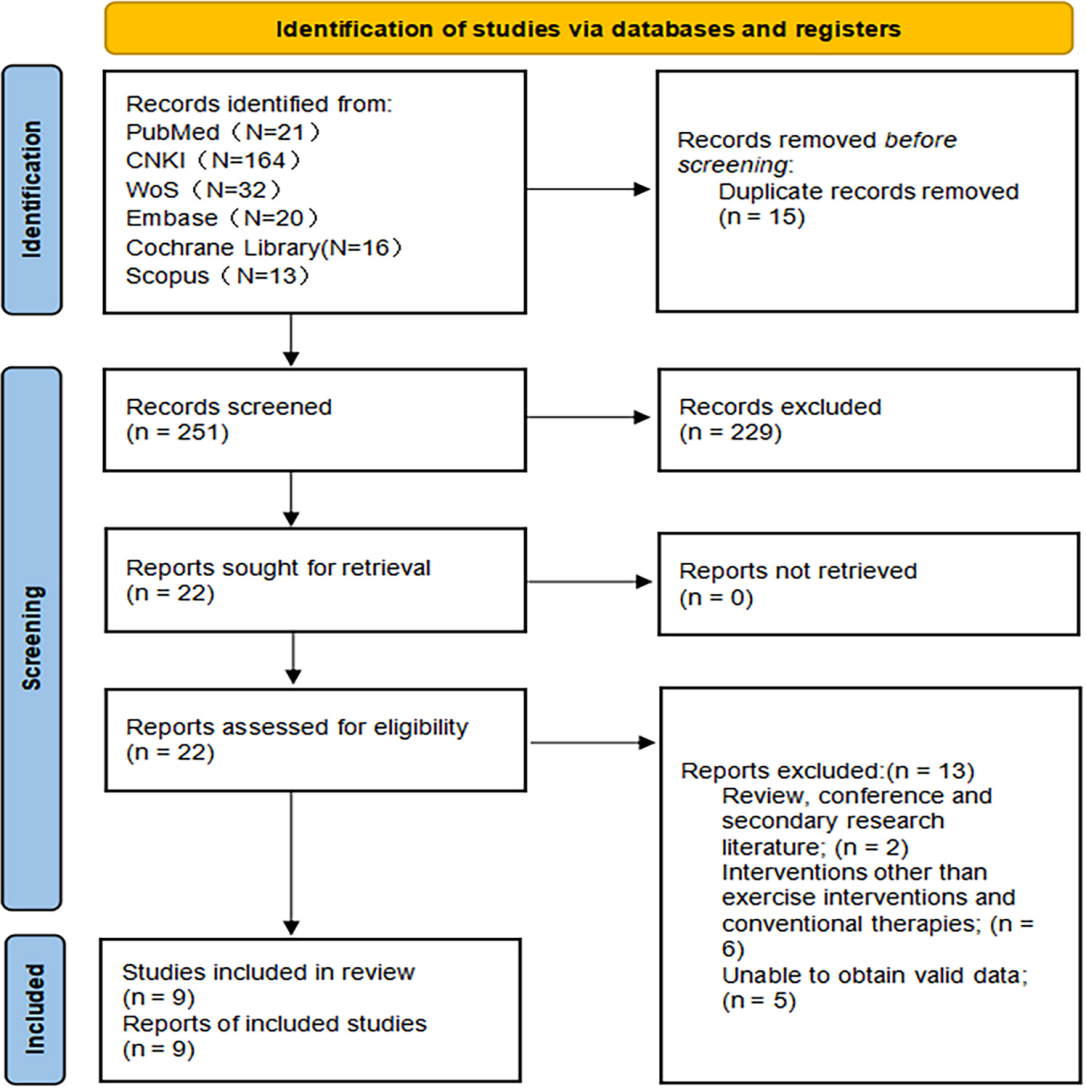
PRISMA flowchart. A total of 266 literature were retrieved, and nine literature were included in the formal analysis.

The initial search yielded 266 potentially relevant records across multiple databases, with the majority retrieved from CNKI (164 records). After a rigorous screening process based on predefined inclusion and exclusion criteria, a total of nine studies were deemed eligible for inclusion in the final analysis, as illustrated in the PRISMA flowchart (Fig. 1). The combined sample size across these studies was 654 participants, including 373 in the experimental groups and 281 in the control groups.

### Overview of included literature

Table 1 presents the basic characteristics of the included studies. Among these, two studies did not report participants’ ages but stated that the subjects were university students. Four studies employed traditional sports activities (*e.g.*, basketball, running, badminton, table tennis) as the intervention, while three studies utilized Tai Chi as the intervention method for the experimental group. In six studies, the control groups received no intervention during the experimental period, whereas the remaining studies implemented placebo-like interventions, such as sitting in silence or receiving psychological counseling.

**Table 1:** Basic information on the included literature.

**First author and year of publication**	**Sample size E/C^a^**	**Gender** **M/F^a^**	**Age** **E/C**	**Intervention (E)**	**Intervention (C)**	**Intervention duration** **(Weeks)**	**Intervention frequency (E)**	**Intervention time or intensity (E)**	**Outcomes**
Yang & Zeng (2017)	26/26	25/27	19.6 ± 1.2/ 19.7 ± 1.4	Tai Chi	Non-intervention	16	4 times per week	60 min each time	CIAS^a^
Chen et al. (2024)	12/12	12/12	19.7 ± 0.76/ 19.9 ± 1.68	Exergames	Pseudo stimulation (2 times per week and 20 min each time)	4	Unreported	60–80% VO_2max_	SAS-C^a^
Gao, Sun & Xiao (2012)	35/34	36/33	Unreported (undergraduate student)	Cycling and Running	Sit in silence (5 times per week and 90 min each time)	8	5 times per week	Heart rate 130–150 beats/min	IAS^a^
Zhang et al. (2024)	30/30	24/36	20.03 ± 0.556/ 20.20 ± 0.610	Aerobic Exercise (running, badminton, table tennis, rope skipping, and gymnastics)	Non-intervention	8	3 times per week	60 min each time	SAS-SV^a^
	30/30	24/36	20.10 ± 0.759/ 20.20 ± 0.610	Tai Chi	Non-intervention	8	3 times per week	60 min each time	SAS-SV
Wen & Chen (2020)	40/40	0/80	20.63 ± 2.06/ 20.34 ± 1.24	Nutritional interventions and short-term high-intensity training	Non-intervention	8	4 times per week	85%–100% maximal heart rate, 4 min and 50%–60% maximal heart rate, 3 min. Total 4 groups.	CIAS
Li et al. (2014)	27/24	51/0	15.41 ± 1.47/ 15.62 ± 1.78	High-intensity interval training	Non-intervention	10	3 times per week	60–70 min each time	IAS
Liu, Liu & Wang (2022)	31/34	Unreported^b^	Unreported (undergraduate student)	Basketball	Non-intervention	10	5 times per week	60 min each time	MPAI^a^
	31/34	Unreported^b^	Unreported (undergraduate student)	Baduanjin	Non-intervention	10	5 times per week	60 min each time	MPAI
Zhang et al. (2023)	31/31	24/38	20.03 ± 0.55/ 20.23 ± 0.62	Conventional exercise (running, basketball, football, badminton, table tennis, and other exercises)	Non-intervention	8	Unreported^b^	60 min each time	IAT^a^
	31/31	24/38	20.10 ± 0.75/ 20.23 ± 0.62	Tai Chi	Non-intervention	8	Unreported^b^	60 min each time	IAT
Zhao, Hao & Jing (2021)	49/50	62/37	20.14 ± 1.27/ 20.21 ± 1.19	Psychological counseling + Moderate-intensity aerobic exercise	Psychological counseling (1 times per week and 120 min each time)	10	2 times per week	Heart rate 130–170 beats/min.	SAS-C

**Notes.**

a E, Experimental group; C, Control group; M, Male; F, Female; CIAS, Chinese Internet Addiction Scale; SAS-C, Smartphone addiction scale-Chinese Version; IAS, Internet Addiction Scale; SAS-SV, Smartphone Addiction Scale-Short Version; MPAI, Mobile Phone Addiction Index; IAT, The Internet addiction Test.

b Some studies did not report complete demographic information or intervention conditions. Where such data were unavailable in the original articles, they could not be included in the table.

Regarding intervention frequency, six studies reported administering interventions three to five times per week. Two studies did not specify the weekly frequency, and one study applied two sessions per week for the experimental group and one session per week for the control group. As for intervention intensity, six studies quantified it based on the duration of each session, typically lasting 60 min. The remaining studies assessed intensity using physiological indicators such as heart rate or maximal oxygen uptake (VO_2_max).

### Assessment of risk of bias

**Figure 2:**
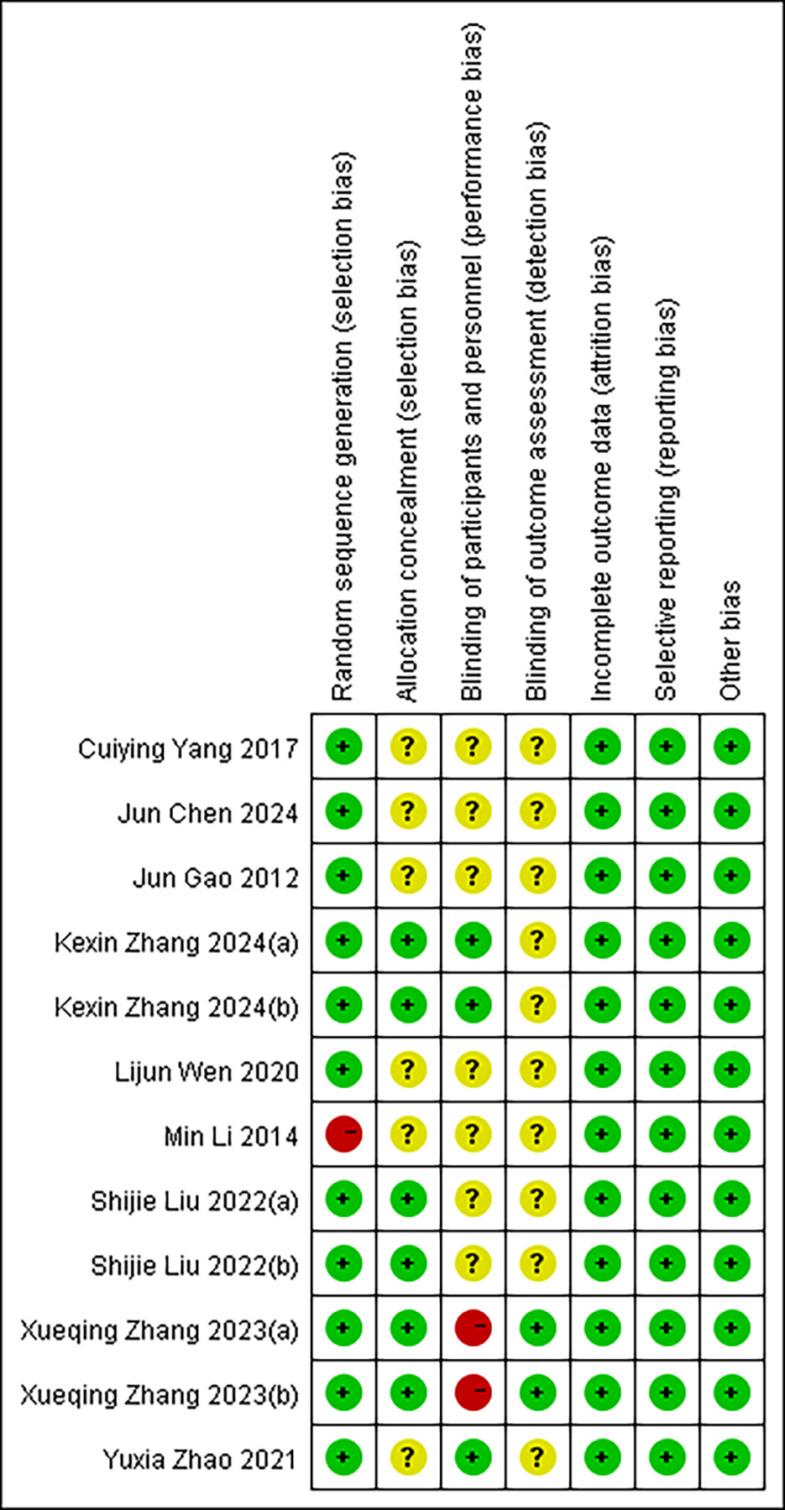
Risk of bias in the included literature. Red means high risk, yellow means unknown risk, and green means low risk. KexinZhang2024(a) (Zhang et al., 2024) used aerobic exercise as an intervention; KexinZhang2024(b) (Zhang et al., 2024) used tai chi as an intervention. Shijie Liu2022(a) (Liu, Liu & Wang, 2022) used basketball as an intervention; Shijie Liu2022(b) (Liu, Liu & Wang, 2022) used baduanjin as an intervention. Xueqing Zhang2023(a) (Zhang et al., 2023) used conventional exercise as an intervention; Xueqing Zhang2023(b) (Zhang et al., 2023) used tai chi as an intervention. KexinZhang2024(a) (Zhang et al., 2024) and KexinZhang2024(b) (Zhang et al., 2024) indicate different intervention groups within Zhang et al. (2024); Shijie Liu2022(a) (Liu, Liu & Wang, 2022) and Shijie Liu2022(b) (Liu, Liu & Wang, 2022) indicate different intervention groups within Liu, Liu & Wang (2022); Xueqing Zhang2023(a) (Zhang et al., 2023) and Xueqing Zhang2023(b) (Zhang et al., 2023) indicate different intervention groups within Zhang et al. (2023). Notes: Yang & Zeng, 2017; Chen et al., 2024; Gao, Sun & Xiao, 2012; Zhang et al., 2024; Wen & Chen, 2020; Li et al., 2014; Liu, Liu & Wang, 2022; Zhang et al., 2023; Zhao, Hao & Jing, 2021.

Figure 2 provides a comprehensive assessment of the risk of bias in the included studies. Regarding random sequence generation, one study was judged to be at high risk due to the absence of random allocation procedures for assigning participants to the experimental and control groups. In the domain of allocation concealment, six studies were assessed as having an unclear risk. Similarly, for the blinding of participants and personnel, six studies were rated as unclear risk, and one study was assessed as high risk. With respect to the blinding of outcome assessment, eight studies were classified as having an unclear risk. In all cases where the risk was deemed unclear, this was due to insufficient reporting to determine whether the study met criteria for low or high risk. Notably, all included studies reported complete outcome data. After thorough evaluation, no evidence of selective reporting or other sources of bias was identified in any of the studies.

Although the studies included in this meta-analysis demonstrated good completeness of reported data and no obvious selective reporting or other biases, certain methodological flaws remain. First, random sequence generation and allocation concealment were judged to be at high or unclear risk in several studies, potentially introducing selection bias and undermining baseline comparability between groups. Second, reporting on blinding procedures was often insufficient, particularly for outcome assessment, which may increase the risk of observer bias. The cumulative impact of these potential biases could affect the accuracy of effect-size estimates and may help explain some of the heterogeneity observed in this analysis. Therefore, although the overall risk of bias is manageable, the results of this meta-analysis should be interpreted with caution, especially in contexts where individual studies exhibit lower methodological quality.

### Meta-analysis

**Figure 3:**
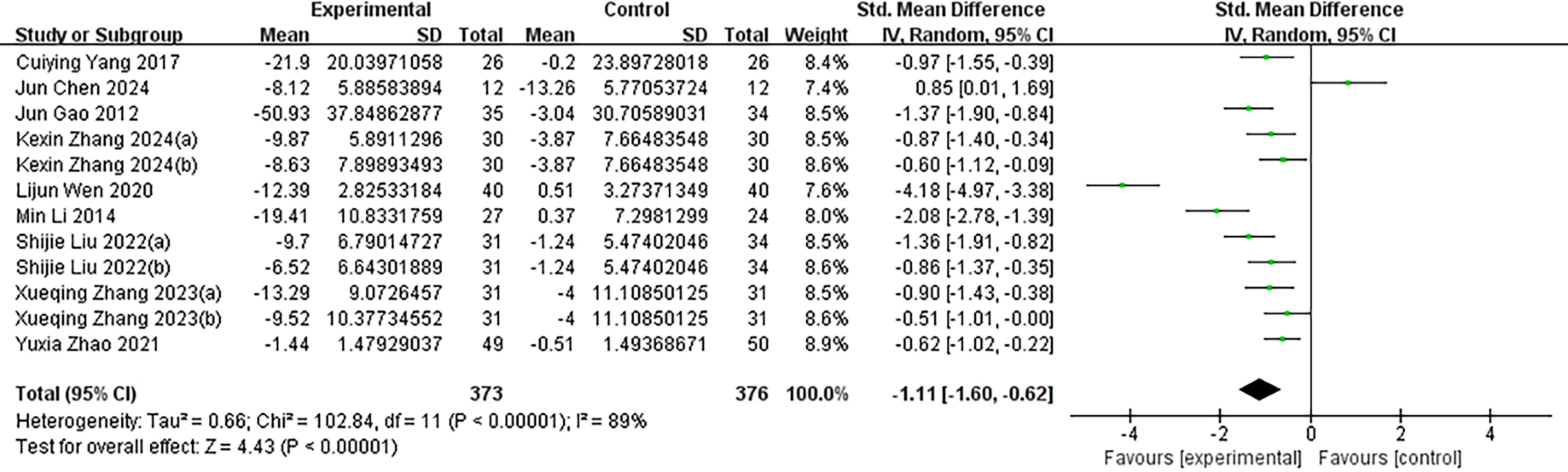
The effectiveness of exercise as an intervention for adolescent internet addiction. The forest plot displays the SMD of the studies along with their 95% confidence intervals. The diamonds indicate the overall SMD and its 95% confidence interval, which are determined through the random effects model. *I*^2^ = 89% implies a large heterogeneity. KexinZhang2024(a) (Zhang et al., 2024) and KexinZhang2024(b) (Zhang et al., 2024) indicate different intervention groups within Zhang et al. (2024); Shijie Liu2022(a) (Liu, Liu & Wang, 2022) and Shijie Liu2022(b) (Liu, Liu & Wang, 2022) indicate different intervention groups within Liu, Liu & Wang (2022); Xueqing Zhang2023(a) (Zhang et al., 2023) and Xueqing Zhang2023(b) (Zhang et al., 2023) indicate different intervention groups within Zhang et al. (2023). Zhang et al. (2024)(a) used aerobic exercise as an intervention; Zhang et al. (2024)(b) used tai chi as an intervention. Liu, Liu & Wang (2022)(a) used basketball as an intervention; Liu, Liu & Wang (2022)(b) used baduanjin as an intervention. Zhang et al. (2023)(a) used conventional exercise as an intervention; Zhang et al. (2023)(b) used tai chi as an intervention. Notes: Yang & Zeng, 2017; Chen et al., 2024; Gao, Sun & Xiao, 2012; Zhang et al., 2024; Wen & Chen, 2020; Li et al., 2014; Liu, Liu & Wang, 2022; Zhang et al., 2023; Zhao, Hao & Jing, 2021.

Post-intervention changes in the mean and standard deviation for both the experimental and control groups were calculated to assess the effectiveness of exercise interventions. Data analysis was conducted using Review Manager version 5.4, with a random-effects model employed for statistical inference. As shown in Fig. 3, the pooled effect size indicated that exercise interventions significantly reduced the severity of IA among adolescents. The SMD was −1.11 (95% CI [−1.60 to −0.62]), reflecting a substantial improvement in the experimental group compared to the control group. However, considerable heterogeneity was observed across the nine included studies, with an *I*^2^ value of 89% (*p* < 0.000), indicating substantial variability in the study outcomes.

### Subgroup analysis

**Figure 4:**
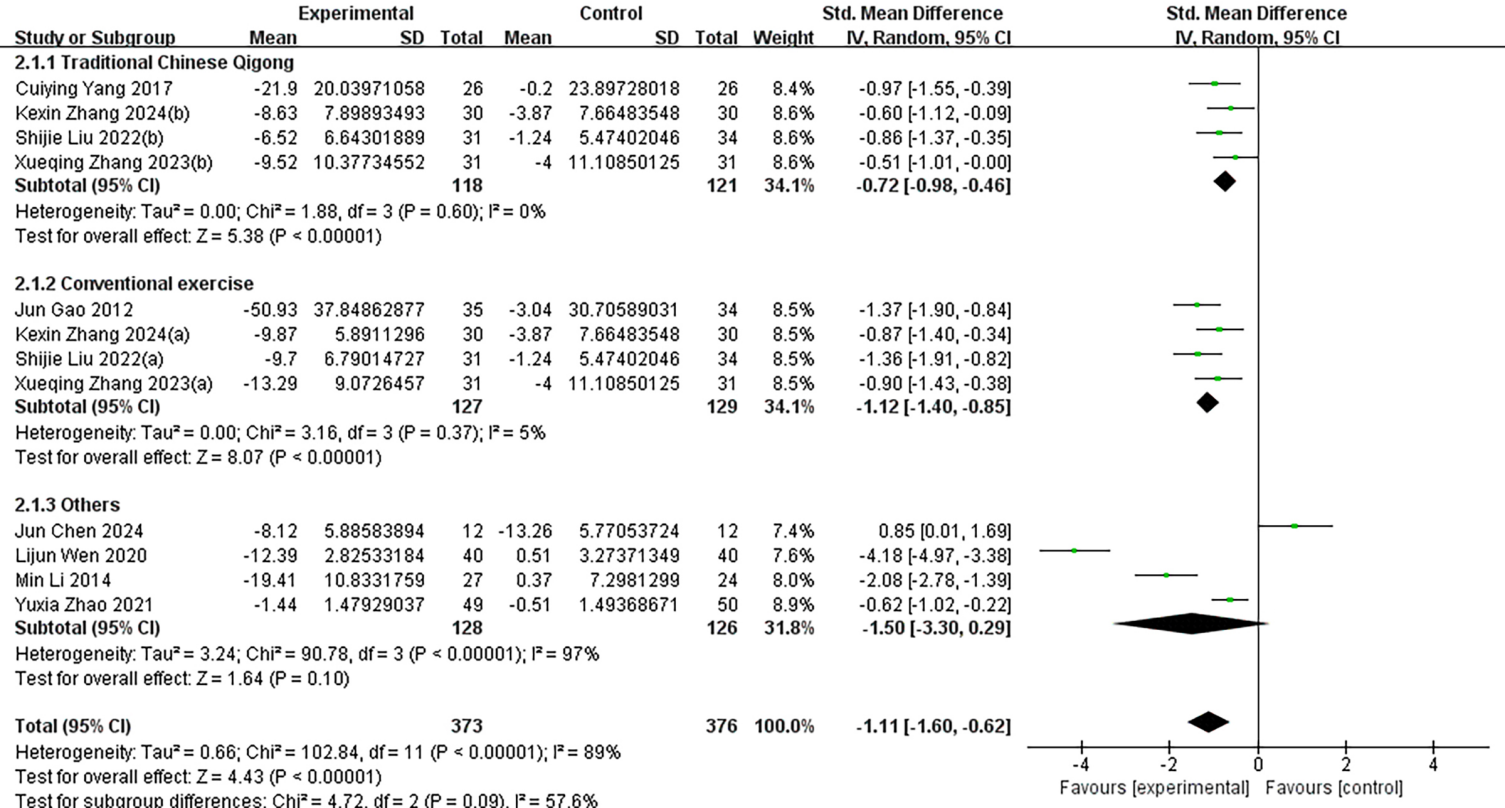
Subgroup analysis of different intervention types in the experimental group. Subgroups were analyzed based on the type of intervention (traditional Chinese qigong, conventional exercise, and other interventions). *I*^2^ = 89% for the total effect implies large heterogeneity. KexinZhang2024(a) (Zhang et al., 2024) and KexinZhang2024(b) (Zhang et al., 2024) indicate different intervention groups within Zhang et al. (2024); Shijie Liu2022(a) (Liu, Liu & Wang, 2022) and Shijie Liu2022(b) (Liu, Liu & Wang, 2022) indicate different intervention groups within Liu, Liu & Wang (2022); Xueqing Zhang2023(a) (Zhang et al., 2023) and Xueqing Zhang2023(b) (Zhang et al., 2023) indicate different intervention groups within Zhang et al. (2023). Zhang et al. (2024)(a) used aerobic exercise as an intervention; Zhang et al. (2024)(b) used tai chi as an intervention. Liu, Liu & Wang (2022)(a) used basketball as an intervention; Liu, Liu & Wang (2022)(b) used baduanjin as an intervention. Zhang et al. (2023)(a) used conventional exercise as an intervention; Zhang et al. (2023)(b) used tai chi as an intervention. Notes: Yang & Zeng, 2017; Chen et al., 2024; Gao, Sun & Xiao, 2012; Zhang et al., 2024; Wen & Chen, 2020; Li et al., 2014; Liu, Liu & Wang, 2022; Zhang et al., 2023; Zhao, Hao & Jing, 2021.

**Figure 5:**
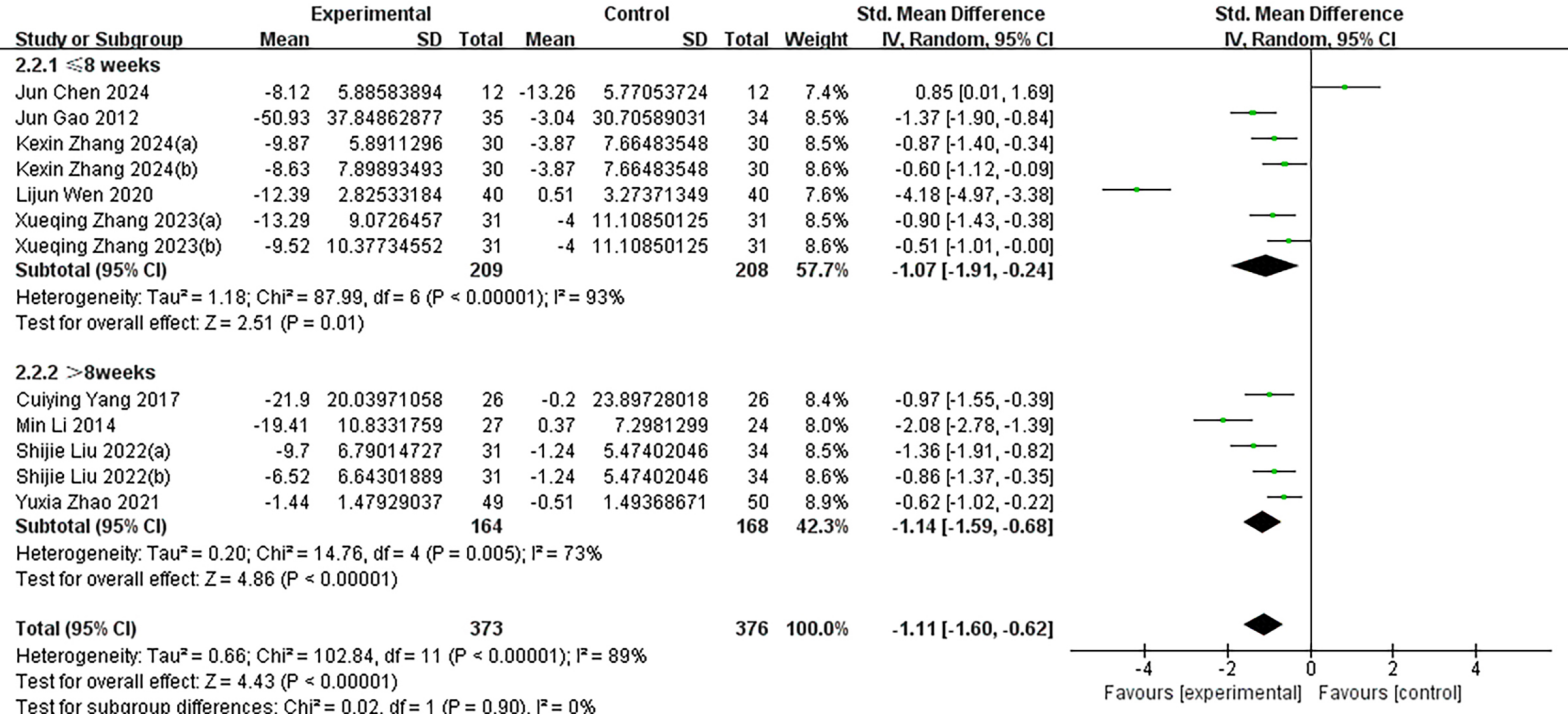
Subgroup analysis of different intervention durations. Subgroup analyses were performed based on intervention duration (≤ 8 weeks or >8 weeks). *I*^2^ = 89% for the total effect implies large heterogeneity. KexinZhang2024(a) (Zhang et al., 2024) and KexinZhang2024(b) (Zhang et al., 2024) indicate different intervention groups within Zhang et al. (2024); Shijie Liu2022(a) (Liu, Liu & Wang, 2022) and Shijie Liu2022(b) (Liu, Liu & Wang, 2022) indicate different intervention groups within Liu, Liu & Wang (2022); Xueqing Zhang2023(a) (Zhang et al., 2023) and Xueqing Zhang2023(b) (Zhang et al., 2023) indicate different intervention groups within Zhang et al. (2023). Zhang et al. (2024)(a) used aerobic exercise as an intervention; Zhang et al. (2024)(b) used tai chi as an intervention. Liu, Liu & Wang (2022)(a) used basketball as an intervention; Liu, Liu & Wang (2022)(b) used baduanjin as an intervention. Zhang et al. (2023)(a) used conventional exercise as an intervention; Zhang et al. (2023)(b) used tai chi as an intervention. Notes: Yang & Zeng, 2017; Chen et al., 2024; Gao, Sun & Xiao, 2012; Zhang et al., 2024; Wen & Chen, 2020; Li et al., 2014; Liu, Liu & Wang, 2022; Zhang et al., 2023; Zhao, Hao & Jing, 2021.

**Figure 6:**
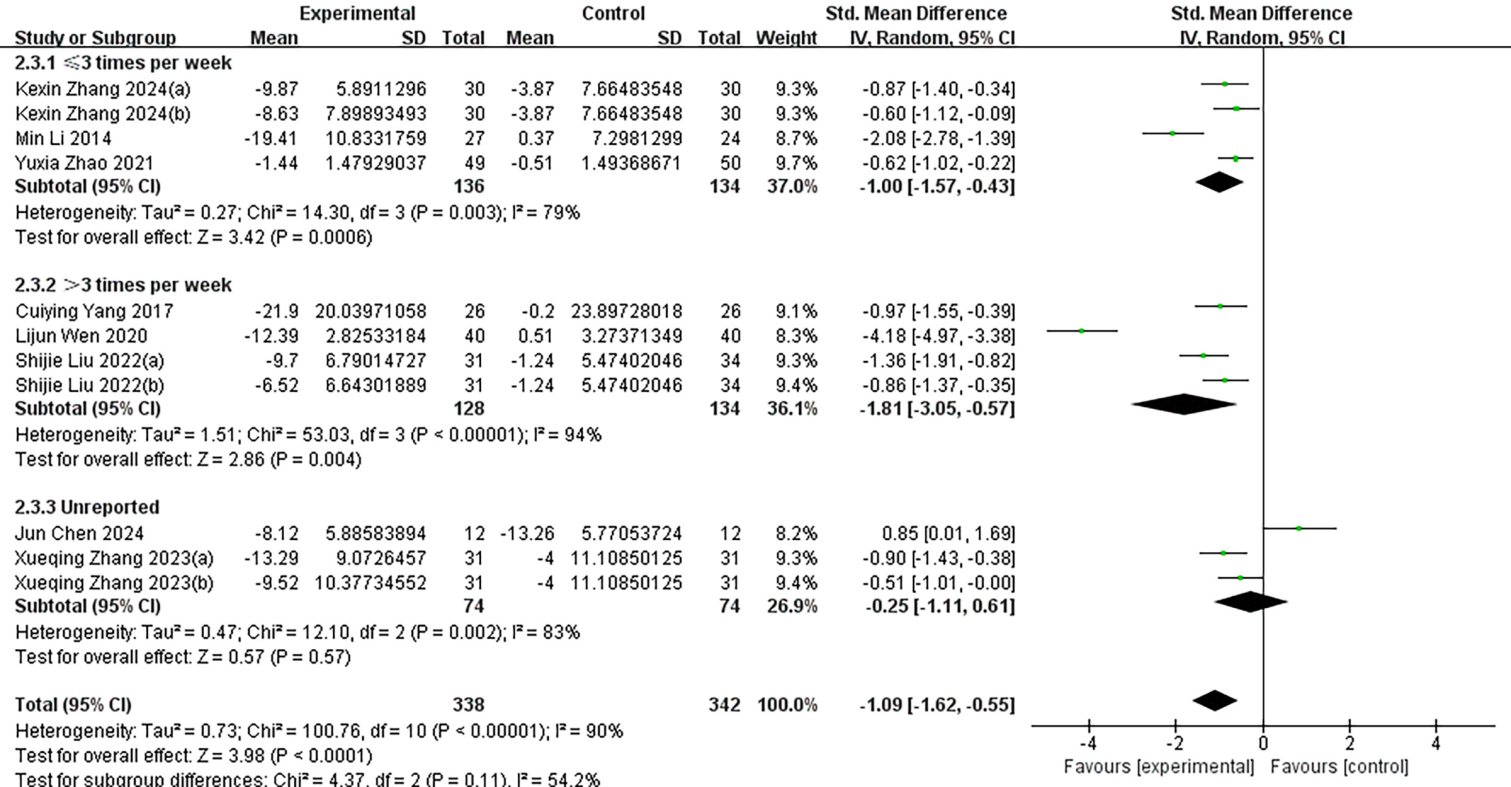
Subgroup analysis of different intervention frequencies. Subgroup analyses were performed based on intervention frequency (≤ 3 times per week, >3 times per week, or unreported). *I*^2^ = 90% for the total effect implies large heterogeneity. KexinZhang2024(a) (Zhang et al., 2024) and KexinZhang2024(b) (Zhang et al., 2024) indicate different intervention groups within Zhang et al. (2024); Shijie Liu2022(a) (Liu, Liu & Wang, 2022) and Shijie Liu2022(b) (Liu, Liu & Wang, 2022) indicate different intervention groups within Liu, Liu & Wang (2022); Xueqing Zhang2023(a) (Zhang et al., 2023) and Xueqing Zhang2023(b) (Zhang et al., 2023) indicate different intervention groups within Zhang et al. (2023). Zhang et al. (2024)(a) used aerobic exercise as an intervention; Zhang et al. (2024)(b) used tai chi as an intervention. Liu, Liu & Wang (2022)(a) used basketball as an intervention; Liu, Liu & Wang (2022)(b) used baduanjin as an intervention. Zhang et al. (2023)(a) used conventional exercise as an intervention; Zhang et al. (2023)(b) used tai chi as an intervention. Notes: Yang & Zeng, 2017; Chen et al., 2024; Gao, Sun & Xiao, 2012; Zhang et al., 2024; Wen & Chen, 2020; Li et al., 2014; Liu, Liu & Wang, 2022; Zhang et al., 2023; Zhao, Hao & Jing, 2021.

**Figure 7:**
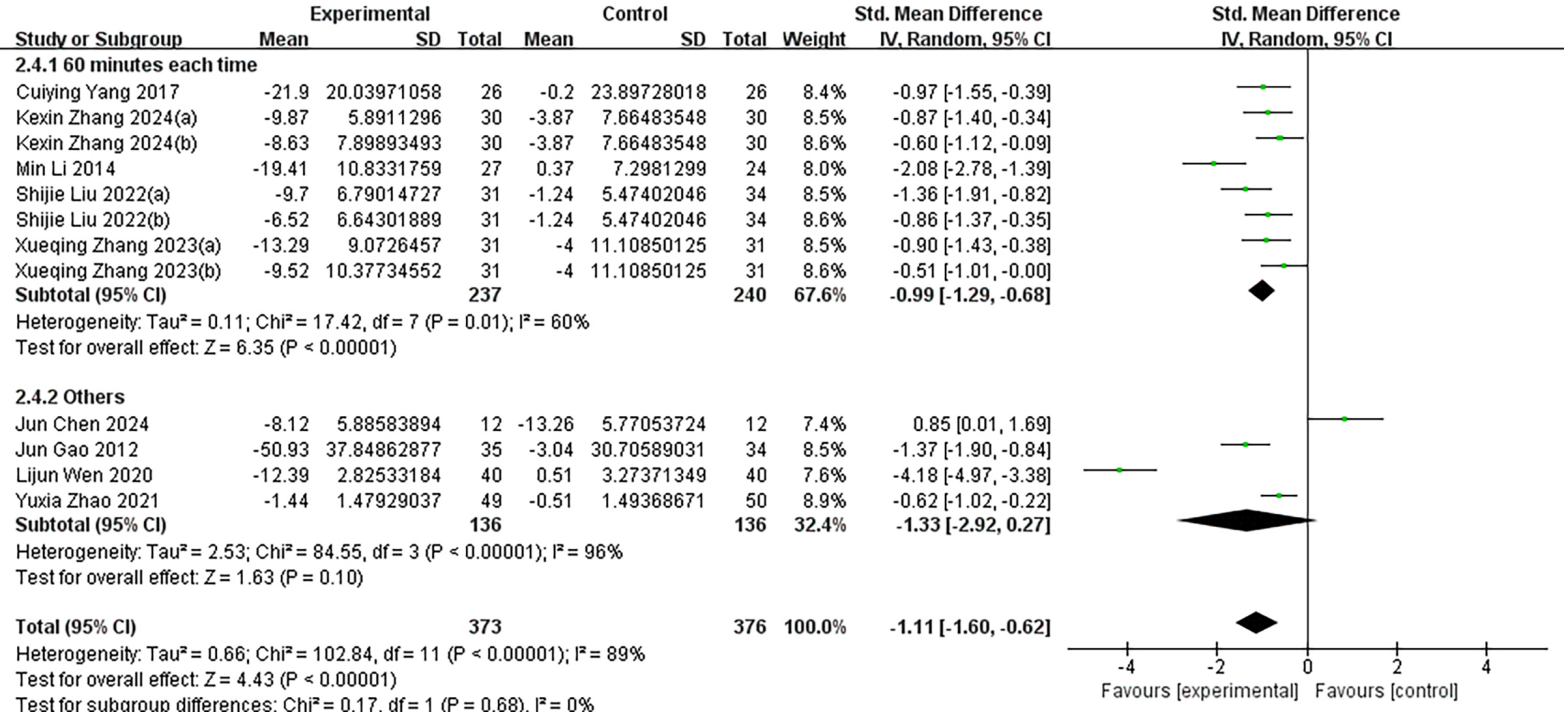
Subgroup analysis of different intervention intensities. Quantified by duration of each intervention or other indicators. *I*^2^ = 89% for the total effect implies large heterogeneity. KexinZhang2024(a) (Zhang et al., 2024) and KexinZhang2024(b) (Zhang et al., 2024) indicate different intervention groups within Zhang et al. (2024); Shijie Liu2022(a) (Liu, Liu & Wang, 2022) and Shijie Liu2022(b) (Liu, Liu & Wang, 2022) indicate different intervention groups within Liu, Liu & Wang (2022); Xueqing Zhang2023(a) (Zhang et al., 2023) and Xueqing Zhang2023(b) (Zhang et al., 2023) indicate different intervention groups within Zhang et al. (2023). Zhang et al. (2024)(a) used aerobic exercise as an intervention; Zhang et al. (2024)(b) used tai chi as an intervention. Liu, Liu & Wang (2022)(a) used basketball as an intervention; Liu, Liu & Wang (2022)(b) used baduanjin as an intervention. Zhang et al. (2023)(a) used conventional exercise as an intervention; Zhang et al. (2023)(b) used tai chi as an intervention. Notes: Yang & Zeng, 2017; Chen et al., 2024; Gao, Sun & Xiao, 2012; Zhang et al., 2024; Wen & Chen, 2020; Li et al., 2014; Liu, Liu & Wang, 2022; Zhang et al., 2023; Zhao, Hao & Jing, 2021.

Given the significant heterogeneity among the included studies, we conducted subgroup analyses to explore potential sources of this variability (Figs. 4–7). Specifically, studies were categorized into three groups based on the type of intervention used in the experimental group.

Traditional Chinese Qigong: This category included Tai Chi and Baduanjin, both of which are traditional Chinese mind–body exercises characterized by regulated breathing, controlled body posture, and focused mental attention. These practices are typically low in intensity, slow in rhythm, and emphasize internal regulation.

Conventional Exercise: This group comprised more commonly practiced moderate- to high-intensity aerobic exercises such as cycling, running, badminton, and table tennis, which emphasize sustained physical activity and external physical load.

Other: This group included integrated or innovative forms of intervention that did not fall into the previous two categories, such as exercise-based games and combinations of moderate-intensity aerobic exercise with psychological counseling.

Subgroup analyses were additionally performed based on intervention duration (≤8 weeks *vs.* >8 weeks), intervention frequency (≤3 times per week, >3 times per week, or not reported), and intervention intensity (measured by the duration of each session or other reported indicators). Despite these efforts, substantial heterogeneity remained within each subgroup, with *I*^2^ values ranging from 89% to 90% (*p* < 0.000), indicating that the selected subgrouping variables did not sufficiently account for the variability in study outcomes. Moreover, comparisons across subgroups revealed no statistically significant differences in effect sizes, suggesting that intervention type and related parameters may not be the primary contributors to the observed heterogeneity.

### Publication bias and sensitivity analysis

**Figure 8:**
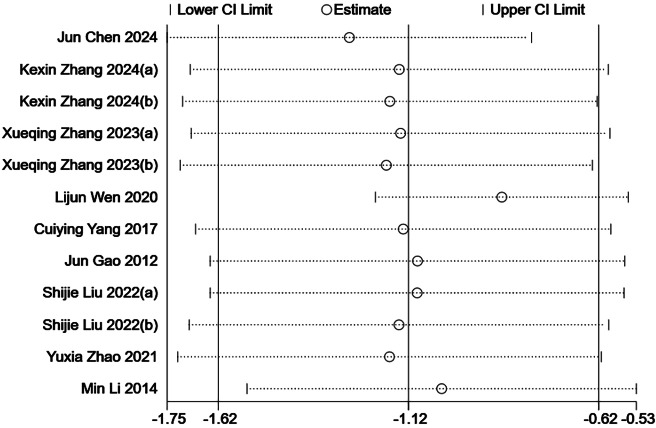
Sensitivity analysis. By excluding each study individually, the overall effect estimates and their confidence intervals do not change much, indicating that the results are robust. KexinZhang2024(a) (Zhang et al., 2024) used aerobic exercise as an intervention; KexinZhang2024(b) (Zhang et al., 2024) used tai chi as an intervention. Shijie Liu2022(a) (Liu, Liu & Wang, 2022) used basketball as an intervention; Shijie Liu2022(b) (Liu, Liu & Wang, 2022) used baduanjin as an intervention. Xueqing Zhang2023(a) (Zhang et al., 2023) used conventional exercise as an intervention; Xueqing Zhang2023(b) (Zhang et al., 2023) used tai chi as an intervention. For three-arm trials, KexinZhang2024(a) (Zhang et al., 2024) and KexinZhang2024(b) (Zhang et al., 2024) indicate different intervention groups within Zhang et al. (2024); Shijie Liu2022(a) (Liu, Liu & Wang, 2022) and Shijie Liu2022(b) (Liu, Liu & Wang, 2022) indicate different intervention groups within Liu, Liu & Wang (2022); Xueqing Zhang2023(a) (Zhang et al., 2023) and Xueqing Zhang2023(b) (Zhang et al., 2023) indicate different intervention groups within Zhang et al. (2023). Notes: Yang & Zeng, 2017; Chen et al., 2024; Gao, Sun & Xiao, 2012; Zhang et al., 2024; Wen & Chen, 2020; Li et al., 2014; Liu, Liu & Wang, 2022; Zhang et al., 2023; Zhao, Hao & Jing, 2021.

To assess potential publication bias, we conducted Egger’s test, which yielded a *p*-value of 0.226 with a 95% confidence interval ranging from –16.59 to 4.42. This result suggests that there is no statistically significant evidence of publication bias among the included studies. However, as with any meta-analysis, the possibility of undetected bias due to small-study effects or unpublished negative results cannot be completely ruled out. Sensitivity analysis using the leave-one-out method revealed that the exclusion of any single study did not substantially affect the 95% confidence interval, thereby confirming the robustness and stability of the overall findings (Fig. 8).

## Discussion

This study conducted a systematic review and meta-analysis to examine the effects of physical exercise interventions on internet addiction among adolescents. The findings indicate that physical exercise significantly reduces the severity of internet addiction in this population, demonstrating a clear positive effect. Statistical analyses showed that the overall effect size of exercise interventions reached the threshold for a “large effect” (Cohen, 1988), highlighting the substantial potential of physical activity as an effective strategy for reducing internet addiction behaviors in adolescents.

Internet addiction is commonly characterized by impairments in both psychological and behavioral functioning, making it not only a mental health disorder but also a broader social concern. Various theoretical models have been proposed to explain the underlying mechanisms of internet addiction. For instance, the Uses and Gratifications Theory and Self-Determination Theory posit that individuals who are motivated by specific psychological needs may engage in excessive use of social media. In this context, social gratification and the fulfillment of psychosocial needs have been identified as strong predictors of social media addiction (Casale & Fioravanti, 2018; Ryan & Deci, 2000). Similarly, the Theory of Planned Behavior suggests that individuals’ positive attitudes toward social media, along with perceived social norms, can predict their likelihood of developing social media addiction, whereas a high level of perceived behavioral control may reduce the risk of engaging in addictive behaviors (Ho, Lwin & Lee, 2017). Notably, theories grounded in the learning perspective—such as classical conditioning (Pavlov, 2023), operant conditioning (Skinner, 1938), and the stimulus–response–reinforcement framework (Hull, 1943)—propose that internet addiction is a learned behavior, acquired through repeated exposure to specific stimuli. When individuals experience positive emotions or psychological reinforcement during internet use, such responses become conditioned and are more likely to be repeated. These learning-based theories are consistent with neurophysiological models of addiction, which indicate that repeated exposure to highly rewarding stimuli sensitizes the brain’s reward systems, leading to the attribution of incentive salience to addiction-related cues (Sun & Zhang, 2021).

Interestingly, exercise prescriptions share certain similarities with internet use in that both can offer positive interactions and entertainment during participation. However, physical activity triggers the release of endorphins in the brain, which bind to opioid receptors, thereby reducing pain and eliciting pleasurable sensations (Weinstein & Lejoyeux, 2020; Harber & Sutton, 1984). In addition, exercise increases the levels of serotonin and dopamine—neurotransmitters that are closely associated with mood regulation and the experience of well-being. The key distinction lies in the context: internet use occurs in a virtual environment, whereas physical exercise is grounded in the real world and promotes tangible benefits for both physical and mental health. The theoretical foundation of exercise-based interventions is rooted in their potential to substitute for certain experiential aspects of internet use while concurrently enhancing physical and psychological functioning. Accordingly, adolescents who actively participate in physical activity tend to report better physical and mental health outcomes and lower levels of internet addiction. This conclusion is further supported by findings from previous studies (Zhou et al., 2024; Liu, Nie & Wang, 2017).

The analysis revealed substantial heterogeneity among the included studies, indicating that variations across individual studies may complicate the interpretation of the pooled effect size. Although subgroup analyses were conducted to investigate potential sources of heterogeneity—such as intervention type, duration, frequency, and intensity—these efforts did not lead to a meaningful reduction in heterogeneity. This outcome likely reflects the complex interplay of multiple underlying factors commonly encountered in meta-analyses, including variations in study settings, intervention protocols, and methodological approaches.

First, regarding intervention types, lower within-group heterogeneity was observed in the Traditional Chinese Qigong and Conventional Exercise groups, whereas the “Other” interventions group exhibited higher within-group heterogeneity. Overall heterogeneity across all subgroups remained high. This may be attributed to considerable variation in the implementation of specific interventions across studies, even within the same category of intervention. Factors such as differences in how exercise intensity was quantified, variability in the control of exercise setting and scheduling, and inconsistent levels of participant adherence could all contribute to these discrepancies. Moreover, cultural context and the baseline severity of internet addiction among participants may further amplify these variations.

Second, the potential influence of intervention duration and frequency was also reflected in the analysis. Although longer durations (>8 weeks) and higher frequencies (>3 sessions per week) were associated with larger effect sizes, the differences between subgroups did not reach statistical significance. This may be due to individual variability in dose–response relationships, as well as limitations such as the small number of included studies and limited statistical power. Additionally, heterogeneity may have arisen from methodological differences, including variations in outcome measurement tools and statistical approaches (*e.g.*, methods for handling missing data). These technical factors may have contributed substantially to the observed heterogeneity.

In addition to intervention design and methodological factors, the high level of heterogeneity observed in this study may also be influenced by several potential moderating variables. First, the baseline severity of internet addiction among participants may significantly affect the intervention outcomes. Individuals with more severe addiction are more likely to experience notable improvements following exercise interventions, whereas those with milder symptoms may exhibit less pronounced changes. Among the included studies, the baseline severity of internet addiction varied; however, these studies did not conduct stratified analyses based on severity levels, which limited our ability to use this variable in subgroup analyses. Second, comorbid conditions often co-occur with internet addiction, and variations in the composition of these comorbidities may lead to differences in intervention responsiveness. For instance, previous research has shown that deficits in self-control and elevated anxiety levels can moderate the relationship between physical activity and internet addiction (Lu et al., 2019; Liu et al., 2024). Socioeconomic status may also indirectly influence intervention effects by affecting loneliness and alienation (Chen et al., 2025). Other factors, such as parents’ active mediation and attitudes toward information and communication technologies, have also been shown to be associated with adolescent internet addiction (Sun et al., 2021). However, these variables were not reported in the included studies, which restricted further analysis.

This study provides valuable evidence regarding the effectiveness of exercise interventions in reducing internet addiction among adolescents; however, several limitations should be acknowledged. First, in the risk of bias assessment, three studies were rated as high risk, which may have influenced the overall reliability of the meta-analytic findings. Future research should aim to enhance methodological rigor, particularly by improving randomization procedures and implementing appropriate blinding techniques.

Second, although subgroup analyses were conducted to explore potential sources of the observed heterogeneity, the selected variables did not meaningfully reduce heterogeneity. This indicates that other uncontrolled or unmeasured factors—such as cultural context, baseline participant characteristics, and adherence to intervention protocols—may have contributed more substantially to the heterogeneity and warrant further investigation.

Third, the included studies employed a range of scales to assess internet addiction, necessitating the use of SMD as the effect size metric. While appropriate under such conditions, the lack of uniformity in assessment tools may have limited both the interpretability and the generalizability of the findings. Future research should prioritize the development and adoption of standardized, cross-culturally validated instruments to improve data comparability and enhance the robustness of results.

Finally, despite a comprehensive search across multiple databases, the number of studies included in this meta-analysis was relatively small, potentially resulting in limited statistical power. This constraint reduced the ability to examine more granular variables and effect modifiers. To strengthen the evidence base and support practical application, future studies should pursue large-scale, high-quality intervention trials.

##  Supplemental Information

10.7717/peerj.19999/supp-1 Supplemental Information 1 List of raw analysis data

10.7717/peerj.19999/supp-2 Supplemental Information 2 PRISMA Checklist

10.7717/peerj.19999/supp-3 Supplemental Information 3 Rationale for the meta-analysis and the contribution of this study

## Additional Information and Declarations

### Data Availability

The following information was supplied regarding data availability: This is a systematic review/meta-analysis.
