# Supplementary material for: Effectiveness of exercise as an intervention for internet addiction in adolescents: a meta-analysis
Source: PeerJ. 2025 Sep 29;13:e19999. doi: 10.7717/peerj.19999 (PMC12490514; doi:10.7717/peerj.19999)
Supplement: Supplemental Information 1 [file peerj-13-19999-s001.docx]

**S1 List of raw analysis data.**

Table1 The data of Meta analysis

|  | Experimental group | | | Control group | | |
| --- | --- | --- | --- | --- | --- | --- |
| Cuiying Yang 2017 | -21.9 | 20.03971058 | 26 | -0.2 | 23.89728018 | 26 |
| Jun Chen 2024 | -8.12 | 5.885838938 | 12 | -13.26 | 5.770537237 | 12 |
| Jun Gao 2012 | -50.93 | 37.84862877 | 35 | -3.04 | 30.70589031 | 34 |
| Kexin Zhang 2024(a) | -9.87 | 5.891129603 | 30 | -3.87 | 7.664835484 | 30 |
| Kexin Zhang 2024(b) | -8.63 | 7.898934928 | 30 | -3.87 | 7.664835484 | 30 |
| Lijun Wen 2020 | -12.39 | 2.825331839 | 40 | 0.51 | 3.273713488 | 40 |
| Min Li 2014 | -19.41 | 10.8331759 | 27 | 0.37 | 7.298129897 | 24 |
| Shijie Liu 2022(a) | -9.7 | 6.790147274 | 31 | -1.24 | 5.47402046 | 34 |
| Shijie Liu 2022(b) | -6.52 | 6.643018892 | 31 | -1.24 | 5.47402046 | 34 |
| Xueqing Zhang 2023(a) | -13.29 | 9.0726457 | 31 | -4 | 11.10850125 | 31 |
| Xueqing Zhang 2023(b) | -9.52 | 10.37734552 | 31 | -4 | 11.10850125 | 31 |
| Yuxia Zhao 2021 | -1.44 | 1.47929037 | 49 | -0.51 | 1.493686714 | 50 |

Table2 The data of Publication bias analysis

| study | n1 | mean1 | sd1 | n2 | mean2 | sd2 |
| --- | --- | --- | --- | --- | --- | --- |
| Jun Chen 2024 | 12 | -8.12 | 5.885838938 | 12 | -13.26 | 5.770537237 |
| Kexin Zhang 2024(a) | 30 | -9.87 | 5.891129603 | 30 | -3.87 | 7.664835484 |
| Kexin Zhang 2024(b) | 30 | -8.63 | 7.898934928 | 30 | -3.87 | 7.664835484 |
| Xueqing Zhang 2023(a) | 31 | -13.29 | 9.0726457 | 31 | -4 | 11.10850125 |
| Xueqing Zhang 2023(b) | 31 | -9.52 | 10.37734552 | 31 | -4 | 11.10850125 |
| Lijun Wen 2020 | 40 | -12.39 | 2.825331839 | 40 | 0.51 | 3.273713488 |
| Cuiying Yang 2017 | 26 | -21.9 | 20.03971058 | 26 | -0.2 | 23.89728018 |
| Jun Gao 2012 | 35 | -50.93 | 37.84862877 | 34 | -3.04 | 30.70589031 |
| Shijie Liu 2022(a) | 31 | -9.7 | 6.790147274 | 34 | -1.24 | 5.47402046 |
| Shijie Liu 2022(b) | 31 | -6.52 | 6.643018892 | 34 | -1.24 | 5.47402046 |
| Yuxia Zhao 2021 | 49 | -1.44 | 1.47929037 | 50 | -0.51 | 1.493686714 |
| Min Li 2014 | 27 | -19.41 | 10.8331759 | 24 | 0.37 | 7.298129897 |
