# Supplementary material for: Effectiveness of exercise as an intervention for internet addiction in adolescents: a meta-analysis
Source: PeerJ. 2025 Sep 29;13:e19999. doi: 10.7717/peerj.19999 (PMC12490514; doi:10.7717/peerj.19999)
Supplement: Supplemental Information 2 [file peerj-13-19999-s002.docx]

| **Section and Topic** | **Item #** | **Checklist item** | **Location where item is reported** |
| --- | --- | --- | --- |
| **TITLE** | | |  |
| Title | 1 | Identify the report as a systematic review. | 1 |
| **ABSTRACT** | | |  |
| Abstract | 2 | The list of abstracts for PRISMA 2020 has been consulted. | 2 |
| **INTRODUCTION** | | |  |
| Rationale | 3 | This is stated in the introduction | 3 |
| Objectives | 4 | This is described in the introduction. | 5 |
| **METHODS** | | |  |
| Eligibility criteria | 5 | This is described in the Methods section. | 5 |
| Information sources | 6 | Searches were performed on PubMed, WoS, Scopus, Cochrane Library, Embase, and the CNKI. The search was conducted from the time the database was created until July 1, 2024. | 7 |
| Search strategy | 7 | Searches were performed on PubMed, WoS, Scopus, Cochrane Library, Embase, and the CNKI. The search strategy will be uploaded as an attachment | 7 |
| Selection process | 8 | This is described in the Literature screening and data extraction section. | 7 |
| Data collection process | 9 | This is described in the Literature screening and data extraction section. | 7 |
| Data items | 10a | This is described in the Statistical analysis section. | 8 |
|  | 10b | This is listed in Table 1 | Table 1 |
| Study risk of bias assessment | 11 | This is described in the Quality of evidence section. | 13 |
| Effect measures | 12 | This is described in the Statistical analysis section. | 8 |
| Synthesis methods | 13a | This is described in the Basic information on the included literature section. | Table 1 |
|  | 13b | This is described in the Statistical analysis section. | 8 |
|  | 13c | This is described in the Statistical analysis section. | 8 |
|  | 13d | This is described in the Statistical analysis section. | 8 |
|  | 13e | If heterogeneity was large, subgroup analysis was used to explore sources of heterogeneity. | 8 |
|  | 13f | Sensitivity analysis showed that the study results were stable. | 8 |
| Reporting bias assessment | 14 | Egger's test | 8 |
| Certainty assessment | 15 | Sensitivity analysis | 8 |
| **RESULTS** | | |  |
| Study selection | 16a | This is shown in Figure 1 | Figure 1 |
|  | 16b | This is described in the Exclusion criteria. | 6 |
| Study characteristics | 17 | This is listed in Table 1 | Table 1 |
| Risk of bias in studies | 18 | This is described in the Quality of evidence section. | Figure 2 |
| Results of individual studies | 19 | For all outcomes, present, for each study: (a) summary statistics for each group (where appropriate) and (b) an effect estimate and its precision (e.g. confidence/credible interval), ideally using structured tables or plots. | Table 1 |
| Results of syntheses | 20a | For each synthesis, briefly summarise the characteristics and risk of bias among contributing studies. | Table 1 and Figure 2 |
|  | 20b | Present results of all statistical syntheses conducted. If meta-analysis was done, present for each the summary estimate and its precision (e.g. confidence/credible interval) and measures of statistical heterogeneity. If comparing groups, describe the direction of the effect. | 6 |
|  | 20c | Present results of all investigations of possible causes of heterogeneity among study results. | 14 |
|  | 20d | Present results of all sensitivity analyses conducted to assess the robustness of the synthesized results. | 15 |
| Reporting biases | 21 | Present assessments of risk of bias due to missing results (arising from reporting biases) for each synthesis assessed. | Figure 2 |
| Certainty of evidence | 22 | Present assessments of certainty (or confidence) in the body of evidence for each outcome assessed. | 15 |
| **DISCUSSION** | | |  |
| Discussion | 23a | Provide a general interpretation of the results in the context of other evidence. | 15-19 |
|  | 23b | Discuss any limitations of the evidence included in the review. | 19 |
|  | 23c | Discuss any limitations of the review processes used. | 19 |
|  | 23d | Discuss implications of the results for practice, policy, and future research. | 19 |
| **OTHER INFORMATION** | | |  |
| Registration and protocol | 24a | CRD42025632958 | - |
|  | 24b | PROSPERO | - |
|  | 24c | CRD42025632958 | - |
| Support | 25 | There was no funding support for this research | - |
| Competing interests | 26 | No competing interests. | - |
| Availability of data, code and other materials | 27 | The datasets used and/or analyzed during the current study are available from the corresponding author upon reasonable request. | - |

*From:*  Page MJ, McKenzie JE, Bossuyt PM, Boutron I, Hoffmann TC, Mulrow CD, et al. The PRISMA 2020 statement: an updated guideline for reporting systematic reviews. BMJ 2021;372:n71. doi: 10.1136/bmj.n71

For more information, visit: <http://www.prisma-statement.org/>
