# Supplementary material for: Effectiveness of exercise as an intervention for internet addiction in adolescents: a meta-analysis
Source: PeerJ. 2025 Sep 29;13:e19999. doi: 10.7717/peerj.19999 (PMC12490514; doi:10.7717/peerj.19999)
Supplement: Supplemental Information 3 [file peerj-13-19999-s003.docx]

**Supplemental File**

**1. The Rationale for Conducting the Meta-Analysis**

The rationale for conducting this meta-analysis is multifaceted, driven by the growing concern of internet addiction among adolescents and the need for effective interventions. Internet addiction, characterized by excessive and compulsive use of the internet, has significant negative impacts on adolescents' psychological well-being and daily functioning. It is associated with a range of adverse outcomes, including reduced physical health, cognitive impairments, and increased psychological distress such as anxiety and depression. Given the severity of these impacts, there is an urgent need for effective therapeutic strategies to address internet addiction.

Physical exercise has emerged as a promising intervention due to its well-documented benefits for both physical and mental health. Exercise can counteract the physiological mechanisms of addiction by improving neural function, enhancing physical fitness, and regulating neurotransmitter levels. Additionally, exercise can provide psychological benefits such as increased self-esteem and social interaction, which can help mitigate the psychological aspects of internet addiction. However, existing studies on the effectiveness of exercise interventions for internet addiction among adolescents show considerable variability in design, sample characteristics, and evaluation methods, leading to low comparability between results. This variability hinders a comprehensive understanding of the efficacy of exercise interventions and limits their broader implementation in clinical practice.

To address these limitations, a systematic review and meta-analysis are essential to integrate existing research data and provide a comprehensive analysis of the effects of physical exercise on adolescent internet addiction. This approach can help overcome the limitations of individual studies, offering more scientific and standardized theoretical foundations and practical guidelines for interventions. By synthesizing the available evidence, this meta-analysis aims to provide a clearer picture of the effectiveness of exercise interventions, thereby informing healthcare professionals and policymakers on the potential benefits of integrating physical activity into therapeutic strategies for managing internet addiction.

**2. The Contribution That the Meta-Analysis Makes to Knowledge**

This meta-analysis contributes to the existing body of knowledge in several significant ways:

Comprehensive Synthesis of Evidence: This study provides a comprehensive synthesis of the available evidence on the effectiveness of exercise interventions for internet addiction among adolescents. By including a wide range of studies from multiple databases, it offers a more robust and comprehensive analysis than previous systematic reviews and meta-analyses. This comprehensive approach helps to fill gaps in the literature and provides a clearer understanding of the overall effectiveness of exercise interventions.

Standardized Effect Size: The use of standardized mean differences (SMD) allows for a more accurate comparison of effect sizes across different studies, despite variations in measurement units and scales used to assess internet addiction. This standardized approach enhances the interpretability and generalizability of the findings, providing a more reliable estimate of the effectiveness of exercise interventions.

Identification of Heterogeneity: The meta-analysis identifies significant heterogeneity among the included studies, highlighting the complexity of evaluating intervention efficacy. While subgroup analyses based on intervention type, duration, frequency, and intensity did not fully explain the heterogeneity, this finding underscores the need for further research to explore other potential sources of variability, such as cultural differences, baseline severity of addiction, and intervention adherence. This insight is valuable for future research and clinical practice, as it suggests that a one-size-fits-all approach may not be effective for all populations.

Assessment of Publication Bias and Sensitivity Analysis: The study includes a thorough assessment of publication bias using Egger’s test and confirms the stability of the findings through sensitivity analysis. These analyses enhance the reliability of the results and provide confidence in the robustness of the conclusions drawn from the meta-analysis.

Practical Implications: The findings of this meta-analysis have important practical implications for healthcare professionals and policymakers. By demonstrating the significant efficacy of exercise interventions in reducing symptoms of internet addiction, this study provides a strong rationale for integrating physical activity into therapeutic strategies. This can help guide the development of more effective and evidence-based interventions for managing internet addiction among adolescents.
